# Societal determinants of flood-induced displacement

**DOI:** 10.1073/pnas.2206188120

**Published:** 2024-01-08

**Authors:** Jonas Vestby, Sebastian Schutte, Andreas Forø Tollefsen, Halvard Buhaug

**Affiliations:** ^a^Peace Research Institute Oslo NO-0134 Oslo, Norway; ^b^Department of Sociology and Political Science, Norwegian University of Science and Technology, NO-7491 Trondheim, Norway

**Keywords:** flood, displacement, natural hazards, disaster, impact

## Abstract

What explains human consequences of weather-related disaster? Here, we explore how core socioeconomic, political, and security conditions shape flood-induced displacement worldwide since 2000. In-sample regression analysis shows that extreme displacement levels are more likely in contexts marked by low national income levels, nondemocratic political systems, high local economic activity, and prevalence of armed conflict. The analysis also reveals large residual differences across continents, where flood-induced displacement in the Global South often is much more widespread than direct human exposure measures would suggest. However, these factors have limited influence on our ability to accurately predict flood displacement on new data, pointing to important, hard-to-operationalize heterogeneity in flood impacts across contexts and critical data limitations. Although results are consistent with an interpretation that the sustainable development agenda is beneficial for disaster risk reduction, better data on societal consequences of natural hazards are critically needed to support evidence-based decision-making.

## Significance

Floods are a major source of human displacement, but variation in displacement levels across contexts remains poorly understood. Using high-resolution data on flood events worldwide, 2000 to 2018, we analyze the moderating influence of core societal characteristics on displacement. Results reveal that floods have potential to generate much higher displacement numbers in contexts of high population exposure, low level of development, nondemocratic governance, and prevalence of armed conflict. However, these factors contribute little to the models’ ability to accurately predict displacement outcomes on new data, pointing to complex causality and critical data limitations. Further scientific progress in this field would benefit from more systematic data collection and better analytical appreciation of displacement as multidimensional behavior that can both increase and mitigate risk.

Between June and September 2022, torrential monsoon rains compounded by exceptional heat-induced glacier melt resulted in catastrophic flooding across Pakistan that left one-third of the country under water. At least 33 million people are estimated to have been displaced by the floods, which by September 6, 2022 had claimed over 1,300 lives, damaged or destroyed around 1.7 million houses, and left more than 6 million people in need of emergency assistance (1–3). The Pakistan floods are part of a well-documented global trend toward increasing frequency and severity of heavy precipitation events over most land area (4). Projections of future changes in the weather system suggest that the occurrence of the most intense precipitation events may almost double for each 1 °C of additional global warming (5).

Available literature offers important insights into common drivers of vulnerability to environmental hazards (6–10), although key knowledge gaps remain (11–13). Central among these is the need for more systematic understanding of how societal structures and vulnerabilities moderate disaster risk (14, 15). Addressing this research gap, we investigate how core socioeconomic, political, and security conditions shape flood-induced displacement worldwide, building on the notion that displacement represents failure of adaptation (16–18).

## Global Trends in Contemporary Flood Disasters

**Fig. 1.**
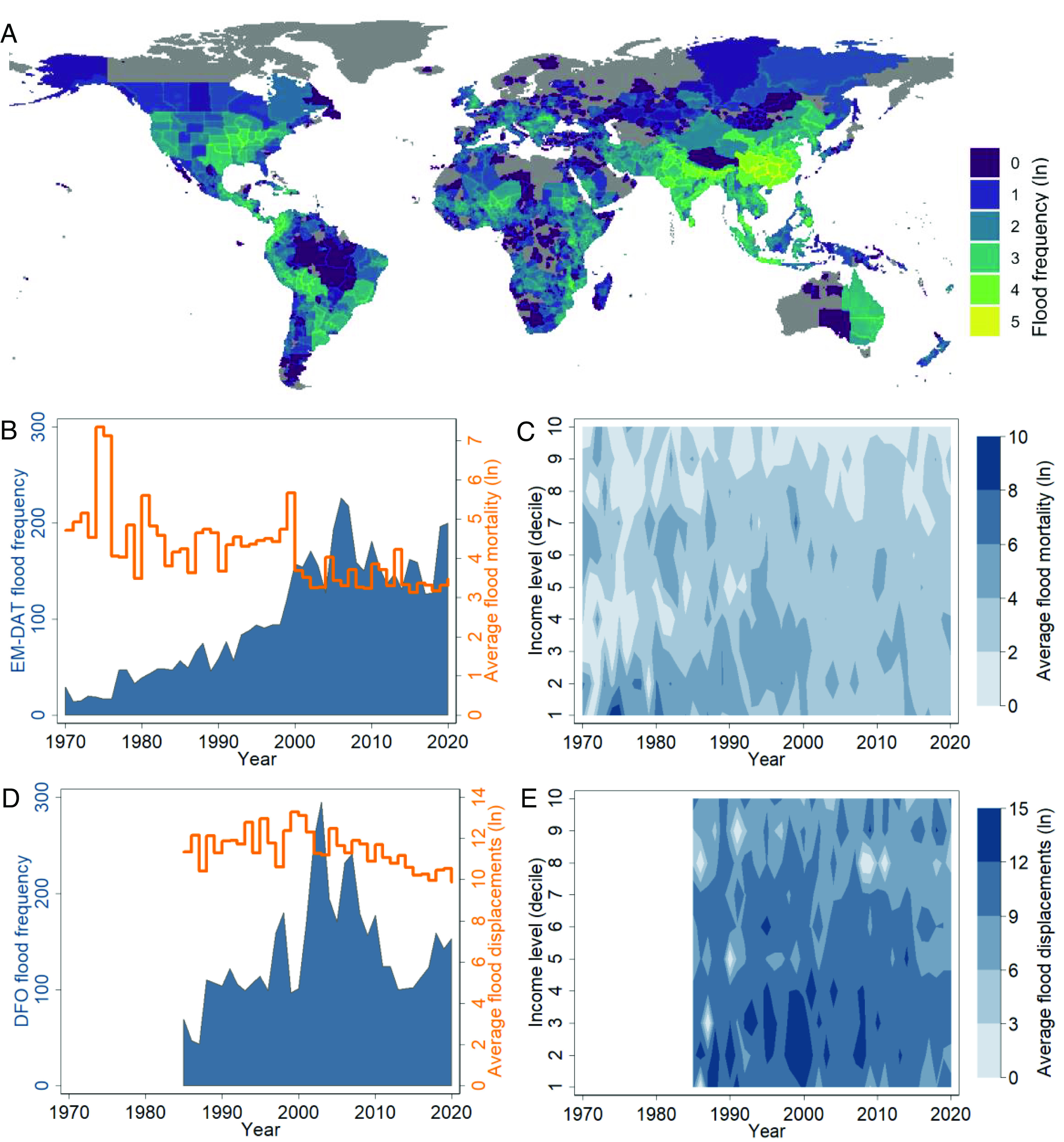
Trends in flood disasters across space and time. (*A*) Frequency of EM-DAT flood disasters by location, 1970 to 2018 (19); gray areas denote regions without any flood disaster in the period [spatial flood extent data from ref. 22 superimposed on a unified grid system (23)]. (*B*) Global frequency and average mortality of EM-DAT flood disasters by year, 1970 to 2020. (*C*) Contour plot of yearly average EM-DAT flood mortality by income group (deciles of GDP per capita in year), 1970 to 2020 (GDP per capita data from ref. 24). (*D*) Global frequency and average displacements of DFO floods by year, 1985 to 2020 (25). (*E*) Contour plot of yearly average DFO flood displacements by income group (deciles of GDP per capita in year), 1985 to 2020. Ln is natural logarithm.

Flood disasters exhibit distinct trends across space and time. Statistics by the International Disaster Database, EM-DAT (19), reveal that flood disasters are especially prominent in South and East Asia, whereas slightly populated polar and dry areas are largely exempt from severe flood risk (Fig. 1*A*).^*^ Over the past 50 y, the global frequency of floods has risen steeply, from an annual average of 25.8 flood disasters in the 1970s to 153.9 disaster events per year on average since 2011 (Fig. 1*B*, blue area). Anthropogenic climate change constitutes a key driver of this growth, with increasing human exposure, land use changes, and improved quality of reporting over time representing other major explanations (20, 21).

In marked contrast to the global surge in flood events, the severity of floods in terms of lives lost is declining. Over the past half century, global average mortality per flood event dropped by nearly 90% (Fig. 1*B*, orange line), from around 272 casualties per disaster in the 1970s (258 floods totaling 70,215 deaths) to 31.5 deaths per event in the last decade (1,539 floods; 48,477 deaths). In the same period, the global population nearly doubled, and growth has been especially high in hazard-prone regions (20).

The remarkable decline in the human cost of floods is evidence of major progress in flood management and disaster risk reduction in high-exposure areas (26–29). Fig. 1*C* documents a negative relationship between level of development and flood mortality. Consistent with earlier findings, it reveals that the deadliest floods concentrate in the lower income deciles, especially in the first half of the period. Average flood mortality for the 10% wealthiest countries remains comparatively low throughout this period.

Mortality rate is not the only relevant metric of flood impact. For every fatality, almost one thousand persons are displaced by flooding according to Dartmouth Flood Observatory (DFO) (25), the leading provider of flood-related statistics. A displaced person in this context is someone who is forced to leave their home or place of residence as a result of disaster (30). Flood displacement can occur both reactively and preemptively, for example as planned evacuation in anticipation of an advancing storm. However, definitions and measurement practices vary (31), implying that available statistic are prone to measurement uncertainty.

Fig. 1*D* displays global trends in floods and displacements since 1985 as recorded by the DFO. Consistent with EM-DAT, the DFO data reveal a peak in flood occurrence in the early 2000s followed by a decline, and the downward trend in displacements matches the falling flood mortality shown in Fig. 1*B*. The contour plot (Fig. 1*E*) reveals that large-scale displacement events concentrate in lower income groups, although the clustering pattern is slightly weaker than for mortality (Fig. 1*C*). However, global average patterns disguise considerable heterogeneity in flood impact across events, between regions, and over time. What explains this variation remains understudied.

## Approach

In order to advance scientific understanding of the conditions under which floods generate widespread human displacement, we draw on the tenets of coupled human and natural systems (32–34). Consistent with this framework, flood-induced risk is conceptualized as a product of complex interactions between natural hazards, human exposure, and societal determinants of vulnerability and resilience that are experienced and play out differently across social scales, from the individual to the nation-state at large. Our analysis focuses on the magnitude of human displacement from distinct flood events as a function of hazard exposure while accounting for prevailing societal contexts at the time of disaster.

**Fig. 2.**
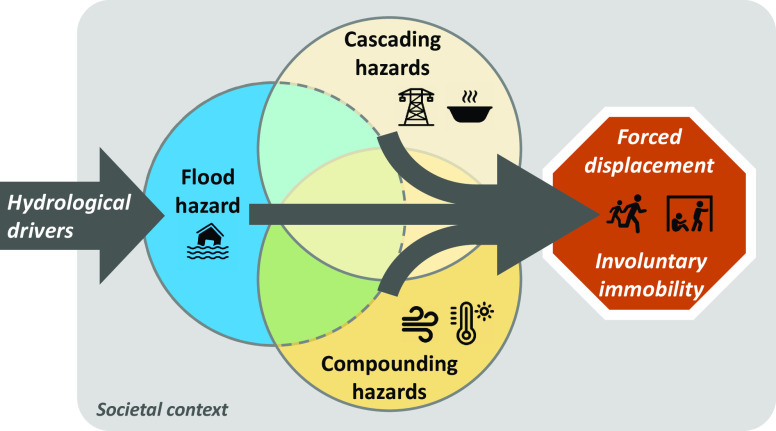
Mobility risks from flooding. Exposure to flood hazard may result in forced displacement or involuntary immobility as a direct result of flooding or through interactive impacts from compounding hazards, such as windstorm or extreme temperatures, and cascading impacts, such as loss of critical infrastructure or compromised food and water security. Key socioeconomic, political, and security contexts play a central role in shaping these interactions and resulting flood-induced mobility risks.

Fig. 2 provides a simple schematic illustration of how these features are related to each other. People directly exposed to flooding as well as those affected by compounding and cascading hazards jointly comprise the pool of latent movers. The subset of exposed people that are forced to flee to avoid or minimize flood-induced harm are classified as displaced persons. Some exposed people may be unable to relocate and are involuntarily immobile while others may decide to stay even when confronted with imminent danger, for example due to strong attachment to place (35), challenging simplistic notions that natural hazards increase individual-level likelihood of displacement in a mechanistic and universal fashion. Common barriers to mobility in the context of disaster include destruction of transportation networks, political restriction on movement, and adverse security conditions (16, 36, 37).

The ratio of exposed to displaced persons will vary greatly across cases, depending on the nature and severity of the flood event (inundation speed and depth, extent of physical destruction, duration of flooding); the prevalence of compounding and cascading hazards within and beyond the flooded areas (severe windstorm, outbreak of diseases, breakdown of services); the preparedness and resilience of the exposed population (disaster management plans, robustness of built infrastructure, early warning systems), as well as contextual factors that may cushion or amplify mobility outcomes (financial resources, government response, armed conflict).

The abrupt and potentially life-threatening nature of severe floods implies that human agency is less central here than for migration in response to gradual environmental change, although aspects of power and privilege may influence the viability and range of mobility options even in the context of rapid-onset disasters (38–40). On the basis of theoretical and empirical relevance, we consider three core contextual dimensions that are expected to affect aggregate displacement levels for a given level of flood exposure. This approach privileges conditions that are amenable to policy input over terrain- and weather-related moderators of the flood–displacement relationship:

(i) All else equal, socioeconomic development should lower the need for disaster-driven mobility. Higher income levels facilitate investing in more comprehensive adaptation schemes, more robust service provision and infrastructure, and better flood protection systems (41, 42). These features are likely to reduce exposure of people and assets to flooding, but they also enable greater opportunities for in situ coping. Although some studies point to low incomes being a barrier to mobility (43, 44), that applies more to high-agency migration than to rapid-onset disaster displacement, which usually is temporary and occurs over short distances (12).

(ii) Good governance likewise should moderate displacement, although this expectation is less unambiguous. Well-functioning and accountable institutions are associated with better protection of vulnerable groups, inclusive urban and rural planning, and more effective disaster preparedness and response, which jointly increase sustainability and resilience (45–47). A more developed civil society also offers greater opportunities for support from nonstate actors in times of crisis. At the same time, democratic governments are more sensitive to domestic audience costs (48) and therefore may be especially likely to initiate inclusive evacuation operations preemptively, potentially inflating overall displacement numbers in an effort to minimize loss of lives.

(iii) A third key contextual factor is security: Armed conflict is an important driver of displacement in its own right (49), but it erodes coping capacity also among those who do not flee the fighting by obstructing markets and commodity flows, destroying assets and livelihoods, and reducing the state’s ability (and willingness) to deliver services and relief aid. The result can be a vicious circle of environmental vulnerability, political violence, and disaster loss (50–52).

A systematic investigation into the empirical merit of these expectations requires, first of all, detailed data on the spatial extent of inundation for each flood event and associated displacement estimates at global scale. The best available data for this purpose are provided by the Global Flood Database (GFD). The GFD is based on the DFO flood event catalogue and provides high-resolution (250 m) raster data of inundated terrain for 913 severe flood events worldwide, 2000 to 2018, based on Moderate Resolution Imaging Spectroradiometer (MODIS) images from NASA’s Aqua and Terra satellites (53). The GFD data represent a subset of all DFO-recorded floods in this period, since adverse meteorological conditions and complex topography sometimes prevent remote sensing. Because many floods transcend national borders, we create unique event-country observations for all floods (N = 1,682) by superimposing the WorldPop population raster data, which contain country ID for each 100-m grid cell. The WorldPop data compare favorably with other population raster data in fine-scale accuracy applications (54), so we replace the GFD flood exposure variable with our own estimates of the number of persons residing in the flooded areas using these data.

Estimates of the number of displaced persons per flood event are provided by DFO, based mostly on news reports and official country statements. Around ¼ of the GFD events (N = 221) lack verified information on the magnitude of human displacement. These observations are dropped from the analysis. DFO does not offer estimates of involuntarily immobile populations. Lack of systematic information on compounding and cascading hazards prevents assessing indirect flood exposure and implications for disaster mobility here.

There are many ways to operationalize socioeconomic, political, and security contexts. We choose to measure these concepts broadly, relying on indicators that are widely used to assess societal progress at global scale. To increase analytical rigor, we combine country-level information on each contextual dimension with corresponding georeferenced variables tapping the situation in the flooded areas. Socioeconomic development is represented by gross domestic product (GDP) per capita as well as a satellite-based index of average nighttime luminosity in the flooded area as proxy for density of local economic activity and infrastructure. The political context is represented by a country-level index of electoral democracy, supplemented by a measure reflecting whether some or all of the flood-exposed population is formally excluded from participation in national politics. To capture the prevailing security situation, we measure the total number of battle-related deaths (BRD) in the country over the previous 10-y period, as well as the total number of BRDs from armed conflict events in the flooded areas during the previous 6 mo. The socioeconomic, political, and security variables jointly speak to the most salient drivers of societal resilience and comprise common sustainability metrics (55).

Lastly, we include a set of flood-specific variables: the number of people directly exposed to flooding; the duration of the flood in days; and a count of the total number of DFO flood events in the country over the previous 10 y to capture accumulated impacts of repeated disasters.

To assess the extent to which these societal conditions moderate flood-induced displacement, we rely on negative binomial (NB) regression analysis with Bayesian estimation techniques, due to their powerful postestimation diagnostics and flexibility in accommodating overdispersed count data. Variable importance and model performance are assessed via both in-sample (2000 to 2014) prediction and out-of-sample (2015 to 2018) cross-validation. Given the complex interplay of multiple hierarchical factors, data limitations, and likely measurement errors for the outcome variable, this is a more robust approach for identifying relevant factors than standard econometric analysis.

In building the statistical models, we first estimate the base model with flood-specific characteristics only (*base*), and then sequentially specify more complex models that additionally incorporate the economic (*e*), political (*p*), and conflict (*c*) components separately and in combination (e.g., *c-e*, *c-p*, *full*). A subset of the models that contain all societal dimensions are limited to country-level (*clvl*) or local-level (*llvl*) predictors. Some models also consider nonlinear tensor spline interactions (*t2*) between the two key flood-specific characteristics since the effect of population exposure on displacement may be nonlinearly related to flood duration and vice versa. Lastly, to better account for heterogeneity in flood characteristics and impacts across regions (cf. Fig. 1*A*), we specify continent-level random intercepts (*ri*) and random slopes for the flood-specific characteristics (*rs*) in most models. See *Materials and Methods* and *SI Appendix* for further details on measurements, modeling approach, and performance evaluation.

## Results

**Fig. 3.**
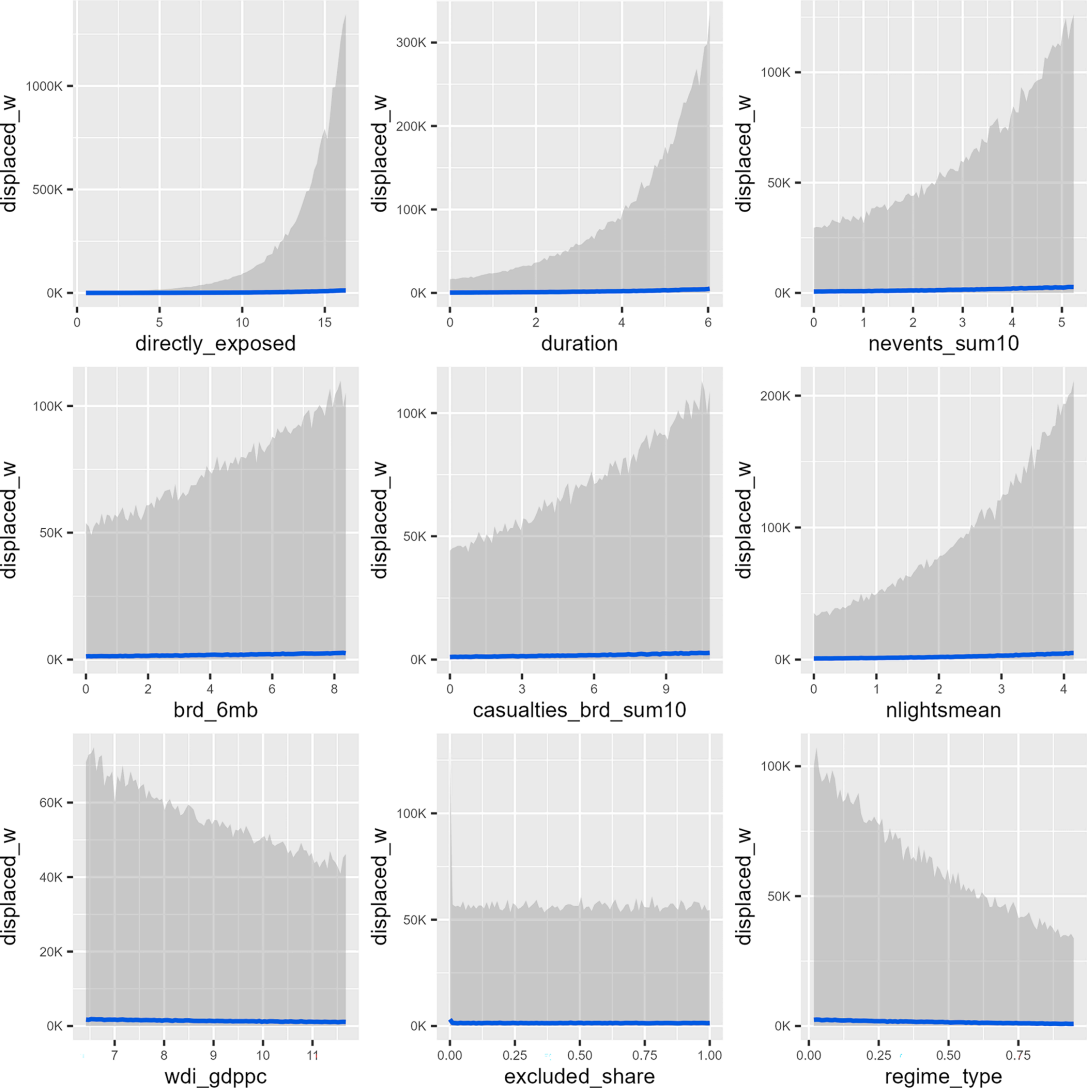
Conditional effect plots for determinants of flood displacement based on the posterior predictive distribution. These plots show the in-sample prediction median (blue line) and the surrounding 80% predictive interval (gray area) across variable inputs holding all other variables at their observed mean, based on the *full+ri+rs* model for the training sample (2000 to 2014), see *SI Appendix*, Table S7.

Fig. 3 shows the estimated in-sample conditional effects from the preferred *full+ri+rs* model on the posterior predictive distribution for each regressor, holding all other variables at their mean (see *SI Appendix*, Table S7 for regression output). The detected functional forms are mostly in line with expectations. Flood displacement is highly influenced by the number of people exposed; the upper bound of the prediction interval rises steeply with increasing population in the flooded areas, as well as with increasing flood duration and extent of previous flood exposure. The latter pattern indicates that flood displacement often is endemic in regions with recurring extreme weather events (e.g., densely populated coastal zones and flood plains in the tropics), and could reflect lack of improvements in flood protection in many disaster hotspots but also increasingly effective early warning systems and life-saving evacuation routines (56).

The socioeconomic indicators reveal that displacement is inversely related to the country’s level of economic development, although the effect is small in substantive terms. At the local level we find a positive association between nighttime light emission and displacement. We interpret this effect as driven partly by population exposure, given the positive correlation between luminosity and local population density, although it also could reflect superior mobility opportunities and evacuation capacity in more urbanized areas. The political variables suggest that democracies are associated with lower levels of displacement than other political systems; the upper bound of the estimated effect entails a reduction in displacement risk for severe floods by two-thirds by shifting from low to high democracy score, holding other factors constant. We also find that floods that affect excluded populations generate lower displacement on average than those that exclusively affect coethnics of the ruling regime. This result breaks with research that finds ethnopolitical discrimination to be an important driver of vulnerability (57, 58), although it may be reflective of lower de facto risk of inundation from flooding in rugged hinterlands where marginalized minority groups often reside (59). As expected, conflict-related casualties—both at the local level and in recent national history—are positively associated with levels of flood displacement.

Collectively, the upper bounds of the predictions reveal meaningful associations between societal characteristics and flood-induced displacement and underscore the relevance of economic development, inclusive governance, and peace for lowering vulnerability to climate-related hazards. That said, we note that the median predictions remain low across the full range of values for all covariates. This reflects the very large spread in observed displacements, even for seemingly comparable contexts, and is representative of the significant challenge of producing accurate predictions of human mobility with macrolevel models (60, 61).

**Fig. 4.**
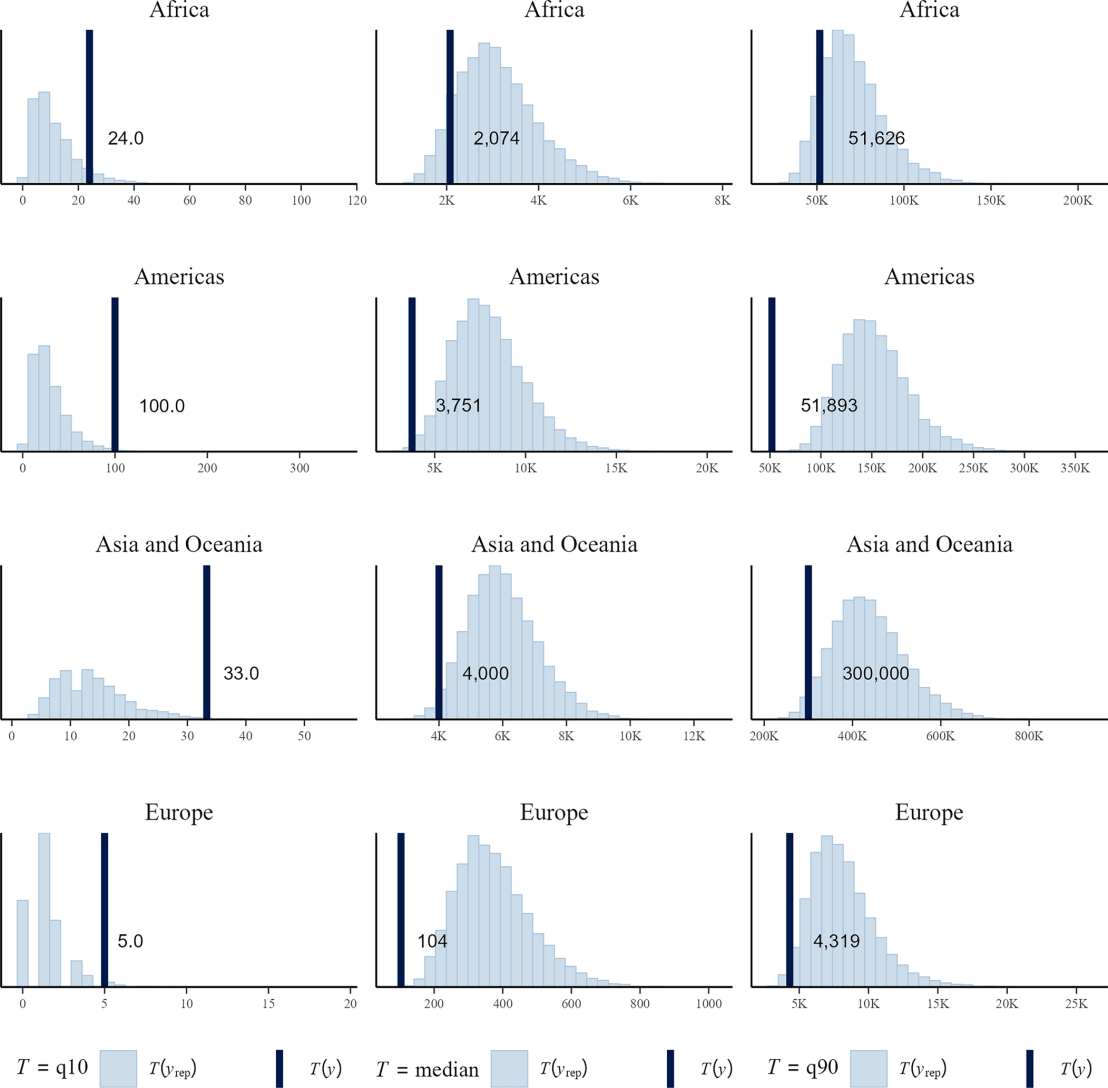
Predictive fit per continent. The plots show the 10th (*Left* column), 50th (*Middle*), and 90th percentile (*Right*) posterior prediction of flood displacement (bars) by continent (rows) based on the *full+ri+rs* model for the training sample (2000 to 2014). Observed 10th, 50th, and 90th percentile numbers of displaced in the training data for each continent are shown by black vertical line.

Fig. 4 shows the calibration of our predictions by comparing the 10th, 50th, and 90th percentiles in-sample prediction based on the *full+ri+rs* model with the observed displacement numbers for the same percentiles for each continent. Despite large differences in the distributions of flood displacements across continents that our model needs to accommodate, the density of the predictions fall quite close to the observed values for most subsamples. Europe is a clear outlier in these data, with median displacement comprising less than 5% of the median observed in the other continents. The 90th percentile event in Europe (4,319 displaced persons) is dwarfed by that in Asia (300,000 displacements). The *full+ri+rs* model tends to overpredict outcomes at higher ranges, especially in the Americas, while underpredicting displacement in the least severe floods. Other model specifications documented in *SI Appendix* (*SI Appendix*, Figs. S5–S7) perform better for in-sample posterior prediction for some regions but are less successful in predicting displacement levels on new data.

### Model Fit and Out-of-Sample Performance.

While the in-sample results appear plausible, the median predictions denote small substantive changes in flood-induced displacement under average conditions. To test whether covariates add predictive value to a model, we estimate a set of models and test their ability to accurately predict the outcome, both in-sample (i.e., using the same data they were trained on) and out-of-sample. We calculate two types of summary statistics from the log posterior predictive density to measure predictive accuracy and compare models. First, we estimate the expected log predictive density (*elpd*) (62). Unlike point metrics such as the mean square error, *elpd* is robust to distributional properties of the outcome (63). Second, we explore model selection and model complementarity using stacking weights (64). The basic idea of stacking is to estimate a weight for each model in an ensemble that minimizes predictive error across observations.

Table 1 shows the *elpd* and stacking weights for the training set (2000 to 2014) and the test set (2015 to 2018), calculated using 15 alternative model specifications. In-sample, the biggest contribution to the prediction comes from adding continent random intercepts (*ri*). Smaller improvements can be made by adding socioeconomic, political, and conflict-related covariates or estimating random slopes for the flood-specific indicators. Although the effects are small in substantive terms, the political covariates (especially democracy) add the most to the prediction, consistent with earlier research on flood mortality (65). The socioeconomic covariates are the least influential in improving model performance in-sample. There is a fair correspondence between the ranking of *elpd* and the stacking weights. In *SI Appendix*, Tables S11–S13, we calculate stacking weights for relevant subsets to further explore the relevance of including different combinations of covariates, as well as testing the validity of the performance estimates through outlier tests.

**Table 1.** Model performance

	In-sample (2000–2014)		Out-of-sample (2015–2018)	
Model	*elpd* LOO	Stacking weight	*elpd* LOO	Stacking weight
*full+ri+rs*	−14,395 (132)	0.14	−2,226 (48)	0.00
*c-p+ri+rs*	−14,407 (133)	0.08	−2,227 (50)	0.16
*llvl+ri+rs*	−14,410 (135)	0.18	−2,222 (48)	0.14
*e-p+ri+rs*	−14,416 (134)	0.09	−2,223 (47)	0.00
*c-e+ri+rs*	−14,419 (135)	0.13	−2,224 (48)	0.00
*p+ri+rs*	−14,424 (134)	0.00	−2,222 (48)	0.12
*clvl+ri+rs*	−14,425 (135)	0.14	−2,226 (49)	0.00
*c+ri+rs*	−14,425 (135)	0.00	−2,226 (49)	0.24
*e+ri+rs*	−14,426 (134)	0.08	−2,222 (47)	0.00
*base+ri+rs*	−14,436 (136)	0.04	−2,222 (48)	0.01
*base+ri*	−14,502 (143)	0.00	−2,219 (47)	0.19
*base*	−14,569 (149)	0.00	−2,226 (46)	0.14
*clvl*	−14,571 (154)	0.10	−2,227 (47)	0.00
*full*	−14,574 (155)	0.00	−2,229 (46)	0.00
*llvl*	−14,577 (152)	0.01	−2,227 (46)	0.00

Note: We measure model accuracy by comparing the posterior predictive distribution to the observed outcome, instead of using a point prediction (such as the mean or median prediction from the model). Accuracy is measured using the expected log predictive density (*elpd*) and stacking weights, estimated using leave-one-out (LOO) cross-validation.

Out-of-sample, the difference between models in terms of *elpd* is small. The *ri* specification remains relevant, but otherwise there is no clear correspondence between the ranks from the training and test samples, implying that the in-sample results perform poorly as indicators of what to expect in the near future. In general, simpler models are preferred out-of-sample.

## Discussion

The empirical analysis uncovered reasonable statistical effects of the contextual variables, broadly in line with expectations. Although the wide heterogeneity in observed displacement levels for a given level of exposure challenges accurate prediction, the analysis was able to establish meaningful upper bounds on the magnitude of flood-induced displacement as a function of shifting socioeconomic, political, and security conditions. These results can be used to indicate plausible worst-case outcomes of flooding in various societal contexts. Controlling for the number of people directly exposed as well as the duration of flooding, the upper limit of the displacement prediction is substantially higher when the flood affects conflict-affected areas in nondemocratic, low-income societies. Ongoing efforts embedded in the Sustainable Development Goals (SDG) agenda toward, inter alia, eradicating poverty and strengthening peaceful and inclusive governance thus also are likely to contribute to lessening future human cost of extreme weather events. Even so, out-of-sample tests demonstrated that the societal indicators struggle to improve our ability to accurately forecast flood-induced displacement on new data. We believe there are both conceptual and analytical reasons for the modest out-of-sample predictive performance.

On the conceptual side, we are confronted with challenges related to legal and operational ambiguity; there is no globally agreed-upon definition of what classifies as a displaced person (and what does not) in terms of primary motive for leaving, required distance traversed, minimum duration of relocation, etc. Accordingly, providers of displacement statistics often must navigate inconsistent, conflicting, patchy, or missing information. The displacement estimates in the GFD data that this analysis relies on originate mostly from media reports, leaving it to the interpretation of the journalist to determine whether and to what extent human mobility within contexts of flooding is newsworthy and portrayed as displacement. In addition, media coverage exhibits considerable geographical variation, with better reporting in urban sites and areas with wire services (66). In the absence of verified information, the GFD sometimes uses estimated numbers of people residing in the flooded areas as a surrogate for displacement, although that could both underestimate and overestimate true displacement.

A related source of uncertainty is the quality of available information. In countries without a free and independent press, information about human consequences of disaster may be deliberately withheld or grossly inaccurate, reshaped by political incentives to inflate or deflate the gravity of the situation. Without transparent reporting mechanisms, such biases are hard to ascertain. Improved penetration of digital information, including social media and remote sensing technologies, provides better coverage over time, but comparisons across societies remain challenging. Satellite imageries are no perfect substitute for high-quality reporting on the ground when studying human impacts of natural hazards.

On the analytical side, extreme precipitation events and surges are sometimes compounded by destructive windstorms that force people to flee across much wider areas than the flooded terrain alone would suggest (67, 68). Uncertainty about the exact location and severity of an approaching storm may motivate inclusive early warnings and preemptive evacuation based on worst-case assessments as a precautionary principle. For example, 375,000 citizens in low-lying parts of New York City were ordered mandatory evacuation ahead of the 2012 Hurricane Sandy, where the resulting flooding ended up displacing “tens of thousands” city residents (69). Depending on whether predisaster evacuation and impacts of compounding and cascading hazards are accounted for, the number of displaced persons registered in the DFO catalogue may be many times higher than the number of residents in the flood-affected area.

**Fig. 5.**
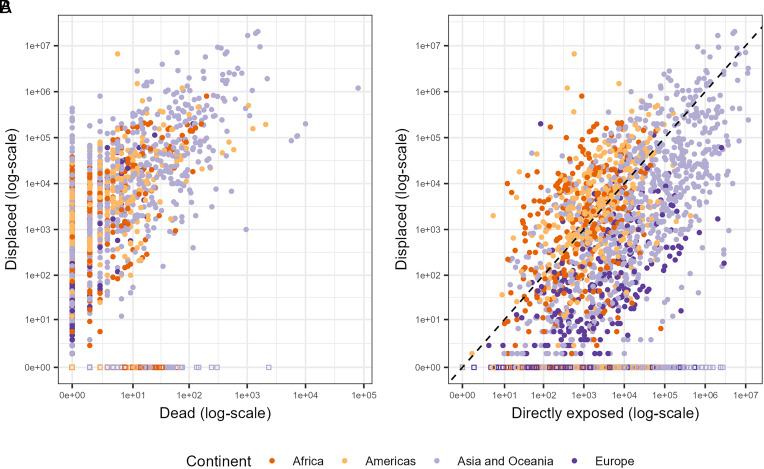
Flood displacements as a function of (*A*) fatalities and (*B*) population exposure by world region. Each dot represents a unique flood-country event. Events with zero displacements reflect missing data and were dropped before analysis. In (*B*), observations above the dashed diagonal have higher reported displacement than estimated population directly exposed to flooding.

We can substantiate this discussion by looking deeper into the GFD data. As shown in Fig. 5*A*, there is a strong, positive correlation between numbers of fatalities and numbers of displacements, but we also detect a significant share of flood events that depart from this general trend. Stacked along the vertical axis are flood events without reported casualties, although some of these had very high displacement figures. Eight nonfatal flood events in the GFD database displaced more than 100,000 persons, three orders of magnitude above the median estimate for nonfatal floods of 161 displacements. Most high-impact events occurred in Asia, but otherwise there is no clear geographical pattern.

Fig. 5*B* compares numbers of displacements with the estimated number of people directly exposed to flooding. Consistent with the importance of population exposure as a predictor of displacement (Fig. 3), the plot reveals a strong positive association between these variables, although with notable spread in displacement for any given exposure level that partly reflects important regional differences. European floods tend to have comparably small shares of displaced to exposed persons, whereas Africa has the highest average ratio. This could indicate a measurement problem, for example that the georeferenced demographic data are less reliable for informal and rural settlements in developing countries (70), resulting in systematic underestimation of people exposed to flood events in many African regions. However, we also believe this pattern is driven by systematic variation in societal sensitivity to climate hazards that is only partly captured by our predictors. For example, tropical regions may be more exposed to compounding hazards since floods often are related to hurricane or cyclone activity. Although a simple comparison of tropical with nontropical floods in the GFD data failed to reveal a consistent difference in relative displacement outcomes (*SI Appendix*, Fig. S1), this issue deserves further scrutiny. In vulnerable contexts, a severe flood also is more likely to generate cascading impacts across sectors, such as breakdown of public services and destruction of critical infrastructure. Such knock-on effects might compel people far beyond the flooded areas to relocate in search of humanitarian assistance. Accounting for local variation in vulnerability at global scale requires better information than what is currently available.

Another analytical reason for the weak out-of-sample results is complex temporal patterns. Global average exposure per flood event rose during the first half of the analysis period but has since dropped, whereas displacement numbers have fallen sharply throughout the period. This implies that the displacement to exposure ratio shifts significantly over time and is considerably lower in the test period than in the training data for the out-of-sample cross-validation. Most of the contextual variables tested here represent relatively inert structural features and are as such much less suited to capture short-term changes in flood impacts than identifying configurations of societal factors that are capable of mitigating adverse effects of flood hazards across longer time scales. However, the poor out-of-sample predictive performance is also indicative of recent progress in flood risk reduction that has occurred much more rapidly than sustainable development more broadly defined. Here, technological innovation, knowledge sharing, and multiscale public–private collaboration have played important roles (71–73).

### Concluding Remarks.

Three broad trends are projected to amplify flood risk in the future: Continuing population growth along coasts, river deltas, and in flood plains; increasing frequency and severity of extreme precipitation events; and sea-level rise. The magnitude of this joint challenge is substantial. A recent study estimates around 50% increase in flood displacement at global scale for every 1 °C of global warming (74). Understanding how structural societal factors may cushion, or amplify, this growing risk is imperative for successful adaptation and sustainable development, but in order to provide relevant insights for decision-making, research but must overcome several nontrivial challenges (75, 76).

Drawing on state-of-the-art remote sensing data on two decades of flood events, we provide evidence that high levels of socioeconomic development, inclusive democracy, and peace are associated with reduced risk of mass displacement in response to human flood exposure. However, our ability to accurately predict displacement levels on new data is constrained by conceptual ambiguity, empirical complexity, and data limitations. Quite likely, our approach underestimates the true human cost of flooding in vulnerable societies, due to higher prevalence of unobserved indirect impacts and lower quality of information in data-poor areas. Further scientific progress in this field is critically dependent on better and more consistent data on natural hazards, human responses, and key features of the local societal contexts.

This research has real-world implications for sustainability also beyond demonstrating a need for improving data collection on disaster vulnerability and impact. Results presented here suggest that enhancing socioeconomic and political conditions (including peace) can help exposed populations overcome natural hazards without large-scale disruptive displacement. That insight aligns well with the SDG agenda and points to important externalities of sustainable development for local coping capacity (77, 78). Yet, human mobility in the context of natural hazards not only is an unwanted impact but also can be an effective coping mechanism. For this reason, numbers of displaced persons can be a misleading indicator of disaster severity, especially where the alternative to widespread displacement would be higher loss of human lives. We welcome further conceptual and analytical progress in understanding disaster mobility as a multidimensional phenomenon that encompasses “normal” risk management behavior as well as submissive, risk-inducing response to natural hazards (79).

In terms of disaster risk reduction, societies thus must take a two-pronged approach that on the one hand entails reducing hazard exposure and on the other enables greater mobility opportunities to manage unavoidable risks. Investing in human and material capital to increase household adaptive and transformative capacity is one important strategy to this end (80), but so are strengthening flood protection and evacuation capacity (81), developing early warning early action programs (82), and supporting equitable, nondiscriminatory governance systems to ensure that no one is left behind (83). Although these and related efforts might not reduce overall levels of displacement in the future, they will increase resilience and ensure that human (im)mobility responses to floods will be safer and occur with greater agency.

## Materials and Methods

The analysis studies georeferenced floods worldwide, 2000 to 2018, as recorded in the GFD (25). The unit of analysis is each distinct flood-country event, represented by high-resolution raster images of the maximum spatial extent of the inundation area, derived from daily 250 m resolution MODIS satellite data. The dependent variable is the number of displaced persons per flood-country event, derived from the related DFO catalogue. Because the displacement estimates originally are aggregated to each flood event, we split the numbers for multicountry floods such that the displacements in each flood-country event reflect the share of the total population exposed to the flood residing in that country. We calculate those directly exposed to flood inundation based on UN-adjusted top-down constrained settlement data from WorldPop (www.worldpop.org), aggregated from 100-m resolution grid cells, calibrated, and overlaid with the GFD flood data. GFD flood events that are listed with 0 displacements are dropped from analysis since 0 in this case reflects lack of verified information.

The statistical models draw on four sets of thematic components. The base model contains two indicators specific to each flood-country event—the number of persons exposed to flood and the duration of flooding in days—and a third country-level flood experience variable, measuring the 10-y moving count of flood events in the country, based on the DFO data. The socioeconomic component contains a calibrated index of average nighttime luminosity in the flooded area based on satellite imageries (84) and country-level GDP per capita (85). The political component includes a dichotomous measure indicating whether any share of the flood-exposed population is formally excluded from national politics, using geo-referenced data from the Ethnic Power Relations Dataset (86), supplemented by level of democracy in the country, measured using the Varieties of Democracy electoral democracy index (87). The conflict component contains a count of the number of BRD from armed conflict events within 20 km from the flooded areas during the previous 6 mo, and a complementary conflict exposure indicator counting the total number of BRD in the country over the previous 10-y period. Both estimates are calculated from data provided by the Uppsala Conflict Data Program (88). The socioeconomic indicators are measured in the year prior to each flood event, and all variables except political exclusion and democracy score are log-transformed to improve the linear fit and reduce outlier influence.

### Statistical Inference and Model Specification.

The number of displaced persons per flood-country event is highly overdispersed. The explanatory variables are likely endogenous and their independent effects on the outcome may be difficult to disentangle. Our ability to identify and isolate causal effects therefore is limited. Instead, we focus on predictive performance, and how the inclusion of covariates affects that ability. To this end, we rely on in-sample and out-of-sample prediction using NB regression with Bayesian inference via the Bayesian Regression Models using Stan (BRMS) package in R (89). This statistical approach also allows us to assess and communicate whole posterior distributions with their relevant upper bounds, rather than only point predictions. We posit that the true causal model would predict well, meaning that the true model at least needs to be among the set of models that are found to perform well.

We build sequentially more complex statistical models, beginning with the base model (*base*) and adding the economic (*e*), political (*p*), and conflict (*c*) components individually and then jointly (e.g., *c-e*, *c-p*, *full*). To account for heterogenous geographical patterns, some models include continent-level random intercepts (*ri*) to represent different baselines in flood impacts across world regions. Some models additionally have random slopes (*rs*) for the two flood-specific base variables to allow for distinct functional forms of the estimated effects across different levels of population exposed and flood duration.

### Evaluation of Predictive Fit.

To ascertain the fit of our models and evaluate variable importance, we employ a train – test predictive framework, where we withhold one portion of the data from the training of the models. Determined by the coverage of the GFD data, the training set covers the years 2000 to 2014, whereas the test data cover 2015 to 2018. We adopt this approach to simulate the task of using recent historical data to forecast future events. Data limitations hinder a more rigorous cross-validation approach, such as estimating models across several shorter moving train-test windows (e.g., ref. 49).

To evaluate the predictive accuracy of the models, we estimate the expected log predictive density (*elpd*) of the posterior distribution using leave-one-out cross-validation and Pareto smoothed importance sampling (90). The main reason for using this evaluation metric is that the distributional properties of our outcome variable are difficult to evaluate via common point metrics such as the mean square error. The *elpd* is proportional to the mean squared error for normally distributed data with constant variance, where larger values (i.e., values closer to 0) are better.

The stacking weights approach is a more targeted procedure for model comparison and model selection as rankings show whether models are complementary or just better/worse. If one model is good at predicting a certain subset while another is better at predicting another subset, they will split weights. If one model is marginally better than another across all observations, it will get the full weight. As the models produce predictive distributions and not point predictions, we follow Yao et al. (64) and use stacking of predictive distributions, where the leave-one-out predictive density is estimated using Pareto-smoothed importance sampling. The stacking weights are estimated on a set of *K* models and sum to 1. We estimate stacking weights for different sets of candidate models (Table 1 and *SI Appendix*, Tables S11–S14). Conditional effects of input parameters are calculated holding all other inputs at their means. The predictions from which conditional effects are estimated are drawn from the posterior predictive distribution.

## Supplementary Material

Appendix 01 (PDF)

## Data Availability

Replication material (Stata and R formats) have been deposited in Harvard dataverse (91).

## Footnotes

^*^To qualify as a disaster in the EM-DAT database, an event needs to fulfill one or more of the following criteria: At least 10 people killed; at least 100 people affected; declaration of a state of emergency; or call for international assistance (19). According to the same source, a flood is defined as a significant rise of water level in a stream, lake, reservoir, or coastal region.
