# Supplementary material for: Societal determinants of flood-induced displacement
Source: Proc Natl Acad Sci U S A. 2024 Jan 8;121(3):e2206188120. doi: 10.1073/pnas.2206188120 (PMC10801835; doi:10.1073/pnas.2206188120)
Supplement: Supplementary file 1 — Appendix 01 (PDF) [file pnas.2206188120.sapp.pdf]

PNAS

Supplementary Information to

Societal determinants of flood-induced displacement

https://doi.org/10.1073/pnas.2206188120

Authors

Jonas Vestby[1], Sebastian Schutte[1], Andreas Forø Tollefsen[1], Halvard Buhaug[1,2,*]

[1] Peace Research Institute Oslo, Oslo, Norway

[2] Norwegian University of Science and Technology, Trondheim Norway

[*] Corresponding author: halvard@prio.org

TOC

1. Data ... 2
   1.1. Data sources and aggregation procedure ... 2
   1.2. Summary statistics, training set, and test set ... 2
   1.3 Additional exploration of the relationship between flood exposure and displacement ... 6
2. Evaluation of predictive performance and model selection ... 8
   2.1. Regression tables ... 8
   2.2. MCMC convergence ... 15
   2.3. Exploring outliers ... 15
   2.4. Assessing predictive fit ... 16
   2.5. Model selection ... 20
3. References ... 22

1. Data

1.1. Data sources and aggregation procedure

The empirical dataset (1) consists of country-level observations of each flood event with reported number of displacements recorded in the Global Flood Database (GFD), 2000–18 (2, 3). GFD provides 250 m raster data generated from satellite images that depict the maximum spatial extent of each georeferenced flood event. Additional flood-specific information, notably the number of reported displaced persons and the duration of the flooding (*duration*), is derived from the underlying Dartmouth Flood Observatory (DFO) data catalogue. We also use statistics from DFO to calculate the number of floods in each country in the 10-year period preceding the event under observation (*nevents_sum10*).

Flood events often transcend national borders. All such events are split into flood-country events before analysis to enable attaching societal contextual information to each event. For each flood-country observation we calculate the number of people directly exposed to flooding by conducting a spatial overlay between the flood rasters and georeferenced 100 m settlement data from WorldPop (www.worldpop.org). For lack of more accurate information, we then divide the total number of displaced in the flood by the fraction of population exposure in each affected country (*displaced_w*). This is likely to introduce measurement error and attenuate estimated effects for the contextual variables. In that sense, our analysis is a hard test of expectations.

Data on armed conflict exposure, counting the number of battle-related deaths within a 20 km buffer around each flooded area during the six preceding months (*brd_6mb*) and the number of battle-related deaths in the country during the preceding 10 years (*casualties_brd_sum10*), are derived from the Uppsala Conflict Data Program (4, 5). Data on nighttime light emissions (*nlightsmean*) (6) and country-level gross domestic product per capita in constant 2011 international dollars (*wdigdppc*) (7) proxy socioeconomic activity. Data on share of the exposed population that is excluded from national politics is coded from the Ethnic Power Relations (Geo-EPR) project (8), dichotomized in the models documented here, supplemented by an electoral democracy index by V-Dem (9).

1.2. Summary statistics, training set, and test set

Before analysis, we split our data into a training set and a test set, with training data being all recorded floods in the period between 2000 and 2014 and the test set consisting of recorded floods in the period between 2015 and 2018. We do not believe that our observations necessarily come from the same data-generating process, meaning that random sampling into training and test groups could be problematic. Instead, our goal is to simulate a process where we use recent historical data to forecast future events, and then test whether we can use in-sample performance as an indication of improved out-of-sample performance.

Tables S1 and S2 present summary statistics for all variables in the training and test sets, respectively, broken down by geographical continent. Oceania is included in Asia due to few observations.

Table S1: Summary statistics, training data, 2000-2014

**Variable**	**N**	**Continent**			
		**Africa, N = 237**	**Americas, N = 293**	**Asia and Oceania, N = 692**	**Europe, N = 212**
displaced_w	1,434	2,074 (1, 800,000)	3,751 (1, 6,649,704)	4,000 (1, 20,324,465)	104 (1, 200,000)
directly_exposed	1,434	959 (10, 60,481)	2,284 (1, 432,967)	21,272 (14, 11,627,289)	3,768 (3, 2,922,713)
duration	1,434	28 (0, 167)	16 (0, 418)	16 (0, 157)	20 (1, 75)
nevents_sum10	1,434	8 (0, 33)	15 (0, 187)	32 (0, 163)	4 (0, 60)
brd_6mb	1,434	0 (0, 1,744)	0 (0, 3,046)	0 (0, 4,299)	0 (0, 162)
casualties_brd_sum10	1,434	0 (0, 49,706)	0 (0, 6,695)	119 (0, 36,854)	0 (0, 11,482)
nlightsmean	1,434	0.1 (0.0, 13.2)	2.4 (0.0, 22.6)	2.0 (0.0, 62.8)	8.2 (0.0, 43.5)
wdi_gdppc	1,434	2,316 (613, 15,727)	13,334 (1,532, 51,209)	4,451 (1,099, 117,271)	21,053 (4,004, 52,770)
excluded_share	1,434	0.13 (0.00, 1.00)	0.22 (0.00, 1.00)	0.23 (0.00, 1.00)	0.06 (0.00, 1.00)
regime_type	1,434	0.44 (0.09, 0.81)	0.79 (0.24, 0.94)	0.37 (0.02, 0.93)	0.84 (0.22, 0.95)

Note: Cells give median sample score with minimum and maximum values in parentheses.

Table S2: Summary statistics, test data, 2015-2018

**Variable**	**N**	**Continent**			
		**Africa, N = 34**	**Americas, N = 61**	**Asia and Oceania, N = 117**	**Europe, N = 36**
displaced_w	248	1,430<br>(5, 197,539)	1,600<br>(2, 1,500,000)	914<br>(1, 715,272)	12<br>(1, 60,565)
directly_exposed	248	1,802<br>(43, 79,980)	1,526<br>(9, 67,874)	13,868<br>(10, 3,834,225)	2,148<br>(21, 2,523,534)
duration	248	13<br>(2, 43)	14<br>(3, 59)	16<br>(1, 76)	7<br>(4, 52)
nevents_sum10	248	9<br>(1, 30)	13<br>(2, 122)	28<br>(0, 108)	5<br>(0, 23)
brd_6mb	248	0<br>(0, 1,001)	0<br>(0, 1,430)	0<br>(0, 1,404)	0<br>(0, 0)
casualties_brd_sum10	248	26<br>(0, 11,402)	0<br>(0, 1,091)	88<br>(0, 50,271)	0<br>(0, 1,964)
nlightsmean	248	1.8<br>(0.2, 16.2)	3.7<br>(0.3, 14.4)	6.0<br>(0.1, 62.1)	10.0<br>(1.5, 20.6)
wdi_gdppc	248	2,809<br>(660, 10,243)	14,807<br>(1,653, 55,719)	6,145<br>(1,757, 84,704)	23,899<br>(10,970, 60,304)
excluded_share	248	0.02<br>(0.00, 1.00)	0.34<br>(0.00, 1.00)	0.26<br>(0.00, 1.00)	0.08<br>(0.00, 1.00)
regime_type	248	0.46<br>(0.18, 0.65)	0.78<br>(0.24, 0.90)	0.36<br>(0.09, 0.88)	0.69<br>(0.27, 0.88)

Note: Cells give median sample score with minimum and maximum values in parentheses.

In the training and test data, we disregard all observations in the GFD where the recorded number of displaced is set to zero. This is because Dartmouth Flood Observatory, on which GFD is based, use the value zero to indicate that no credible displacement estimate has been recorded. In preliminary tests, we explored ways to replace the missing values via various imputation methods (before model fitting with multivariate imputation by chained equations (MICE) and during model fitting using the capabilities in the brms-package in R), but these approaches failed to produce models that converged.

Table S3 gives some indication of possible bias in the analysis sample by comparing the excluded observations with our valid data (displacement > 0) for three flood-specific dimensions: reported fatalities, size of the affected population, and the duration of the flood event. Unsurprisingly, most floods with missing displacement estimates have zero recorded fatalities and half also have zero estimated population exposure, owing to the modest magnitude and peripheral location of many of these events. Even so, some excluded events affected a high number of people and five observations with missing displacement numbers have death counts above 100 (the highest at 2,344 reported fatalities).

Table S3: Core characteristics of floods w/ versus w/o missing displacement

**Variable**	**dead**		**directly exposed**		**duration**		**Total**
	**0**	**>0**	**0**	**>0**	**0**	**>0**	
**displaced_w**							
**0** (*excluded*)	973 (35%)	153 (5.4%)	583 (21%)	543 (19%)	2 (<0.1%)	1,124 (40%)	1,126 (40%)
**>0**	765 (27%)	917 (33%)	0 (0%)	1,682 (60%)	10 (0.4%)	1,672 (60%)	1,682 (60%)
**Total**	1,738 (62%)	1,070 (38%)	583 (21%)	2,225 (79%)	12 (0.4%)	2,796 (100%)	2,808 (100%)

Note: Cells give number of observations with fractions of each two-by-two matrix in parentheses.

Tables S4 and S5 provide descriptive statistics for the explanatory variables for the training (S4) and test (S5) samples for the observations with missing displacement. Compared to the statistics for the valid samples in Tables S1-S2, it is again clear that the excluded floods are mostly minor, peripheral events (i.e., the numbers of people exposed are low, as are nighttime light emissions).

Table S4: Summary statistics, 2000-2014, data with missing displacement

**Variable**	**N**	**Continent**			
		**Africa, N = 216**	**Americas, N = 178**	**Asia and Oceania, N = 370**	**Europe, N = 209**
displaced_w	973	0 (0, 0)	0 (0, 0)	0 (0, 0)	0 (0, 0)
directly_exposed	973	0 (0, 66,322)	0 (0, 190,426)	36 (0, 2,725,411)	312 (0, 2,414,513)
duration	973	20 (1, 167)	12 (0, 115)	12 (1, 140)	11 (1, 78)
nevents_sum10	973	3 (0, 31)	14 (1, 184)	21 (0, 163)	3 (0, 58)
brd_6mb	973	0 (0, 662)	0 (0, 80)	0 (0, 2,449)	0 (0, 3)
casualties_brd_sum10	973	0 (0, 49,600)	0 (0, 6,695)	0 (0, 23,444)	0 (0, 10,196)
nlightsmean	973	0.1 (0.0, 24.0)	0.7 (0.0, 16.3)	0.7 (0.0, 57.3)	6.3 (0.0, 43.3)
wdi_gdppc	973	2,435 (645, 15,727)	15,032 (1,510, 52,081)	5,703 (1,056, 117,271)	18,560 (3,451, 85,033)
excluded_share	973	0.07 (0.00, 1.00)	0.43 (0.00, 1.00)	0.23 (0.00, 1.00)	0.07 (0.00, 1.00)
regime_type	973	0.34 (0.13, 0.80)	0.79 (0.33, 0.93)	0.35 (0.01, 0.92)	0.81 (0.23, 0.95)

Note: Cells give median sample score with minimum and maximum values in parentheses.

Table S5: Summary statistics, 2015-2018, data with missing displacement

**Variable**	**N**	**Continent**			
		**Africa, N = 28**	**Americas, N = 45**	**Asia and Oceania, N = 51**	**Europe, N = 29**
displaced_w	153	0 (0, 0)	0 (0, 0)	0 (0, 0)	0 (0, 0)
directly_exposed	153	0 (0, 4,636)	0 (0, 23,052)	0 (0, 308,679)	67 (0, 296,480)
duration	153	12 (2, 39)	14 (1, 45)	14 (1, 45)	4 (2, 45)
nevents_sum10	153	5 (0, 18)	14 (2, 122)	26 (0, 108)	4 (0, 22)
brd_6mb	153	0 (0, 149)	0 (0, 3,269)	0 (0, 1,157)	0 (0, 0)
casualties_brd_sum10	153	10 (0, 8,487)	0 (0, 0)	0 (0, 43,259)	0 (0, 5,998)
nlightsmean	153	2.1 (0.2, 11.7)	2.8 (0.0, 23.4)	1.1 (0.0, 62.3)	9.7 (0.0, 25.0)
wdi_gdppc	153	2,775 (660, 16,518)	14,283 (6,444, 55,719)	9,247 (1,767, 84,704)	19,321 (7,678, 39,760)
excluded_share	153	0.08 (0.00, 1.00)	0.70 (0.00, 1.00)	0.14 (0.00, 1.00)	0.20 (0.00, 1.00)
regime_type	153	0.40 (0.15, 0.73)	0.75 (0.31, 0.90)	0.42 (0.09, 0.88)	0.59 (0.27, 0.87)

Note: Cells give median sample score with minimum and maximum values in parentheses.

1.3 Additional exploration of the relationship between flood exposure and displacement

In the article Figure 5B, we explore the relationship between flood exposure and displacement for all events across continents. All plots above the diagonal dashed line denote floods where the reported number of displaced persons exceed the estimated number of people residing in the flooded areas. A plausible explanation would be that these events involved compounding hazards that affected additional people outside the flood zones, although it also could reflect an underestimation of the size of the population exposed to flooding or overreporting of flood-induced displacement. To assess whether devastating impacts from tropical storms could be a driver of 'excess' displacement, Figure S1 visualizes the same association broken down by world region (panels) and flood type. There is no clear pattern in the data to suggest that high displacement to exposed ratios are driven by tropical storms.

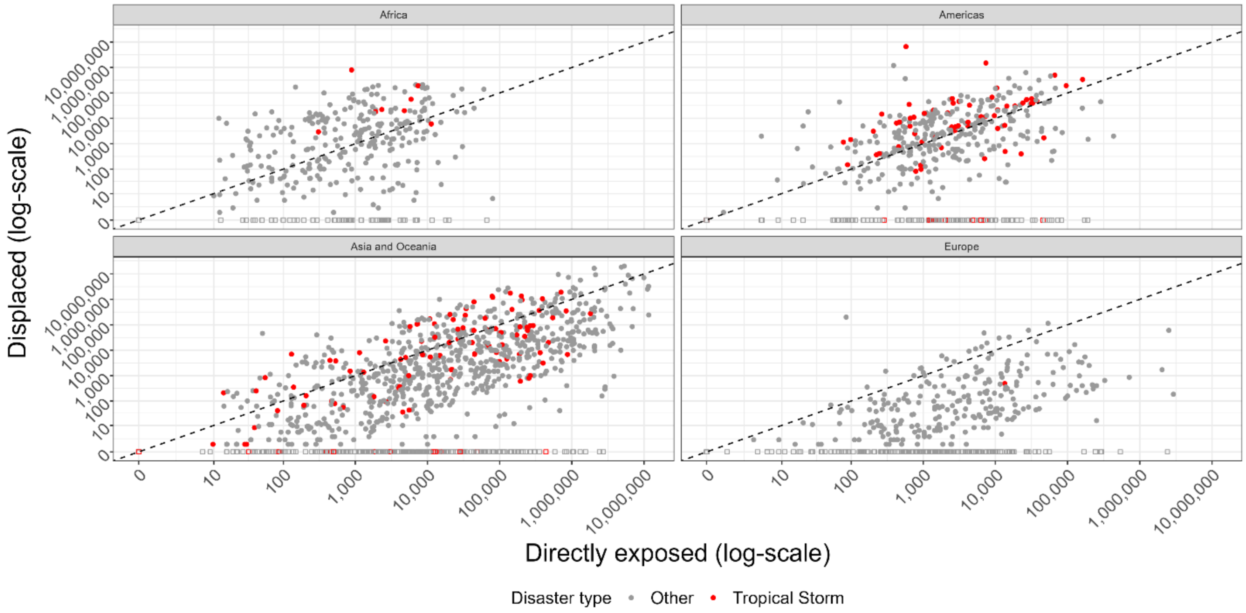

Figure S1: Flood displacements as a function of population exposure by disaster type and continent

2. Evaluation of predictive performance and model selection

2.1. Regression tables

This section presents the results from a parsimonious subset of the best-performing and most interesting Negative Binomial regression models. Complete results for all 19 models are documented and can be reproduced in the replication materials (see also Table S14).

Table S6 shows the most basic specification of the full model (i.e., the model with all socioeconomic, political, and conflict-related indicators in addition to the base flood-specific variables). Table S7 introduces random intercept to better account for systematic variation in flood impact across continents and random slopes for people directly exposed to flood inundation and flood duration to handle heterogenous associations between these factors and displacement. These additions substantially improve model fit and they also make the effects of the contextual variables much clearer (estimates are incidence rate ratios, which should be interpreted multiplicatively). Interestingly, the point estimate for democracy is positive in Table S6 but negative and very significant in Table S7, indicating a Simpson's Paradox, where autocracies have less displacement than democracies in the global sample, but within each continent, democracies have less displacement than autocracies, controlling for other factors (10).

We can also see that the fixed effects are explaining little variance in the outcome (marginal R^2 0.005), while adding the random effects accounts for a substantial share of the variance (conditional R^2 0.197). The intraclass correlation is close to 1, which also suggests that a hierarchical model is very useful (the variance in Y is mostly accounted for by the clustering).

In Table S8, we model people directly exposed to flood inundation and flood duration as a non-linear tensor spline interaction. The fixed effects portion of the model now explains much more variance compared to the random effects portion, although the latter remains important.

Table S6: Full model (full)

	displaced w	
Predictors	*Incidence Rate Ratios*	*CI (95%)*
Intercept	2141.69	428.35 – 11085.79
directly exposed	1.30	1.23 – 1.37
duration	1.97	1.71 – 2.26
nevents sum 10	1.77	1.59 – 1.96
brd 6 mb	1.02	0.95 – 1.11
casualties brd sum 10	1.01	0.97 – 1.06
nlightsmean	1.38	1.12 – 1.69
wdi gdppc	0.65	0.54 – 0.79
Iexcluded_share>0TRUE	1.03	0.75 – 1.41
regime type	1.61	0.91 – 2.82
Observations		1,434
R^2 Bayes		0.075

Table S7: Full model with random intercept and random slopes (full+ri+rs)

	**displaced w**	
Predictors	*Incidence Rate Ratios*	*CI (95%)*
Intercept	369.99	7.22 – 14701.23
directly exposed	1.36	0.76 – 2.11
duration	1.53	0.66 – 3.08
nevents sum 10	1.34	0.65 – 2.60
brd 6 mb	1.09	1.02 – 1.17
casualties brd sum 10	1.09	1.05 – 1.13
nlightsmean	1.55	1.30 – 1.84
wdi gdppc	0.91	0.75 – 1.09
Iexcluded_share>0TRUE	0.43	0.32 – 0.59
regime type	0.32	0.18 – 0.55
Random Effects		
σ^2		573293491073.70
τ_{00}		24437872923.78
ICC		0.97
N $_{continent}$		4
Observations		1,434
Marginal R^2 / Conditional R^2		0.005 / 0.197

Table S8: Full model with random intercepts and tensor splines (full+ri+ t2)

	**displaced w**	
Predictors	*Incidence Rate Ratios*	*CI (95%)*
Intercept	28337.47	1879.69 – 365488.06
nevents sum 10	1.47	1.33 – 1.62
brd 6 mb	1.09	1.02 – 1.17
casualties brd sum 10	1.10	1.06 – 1.15
nlightsmean	1.60	1.36 – 1.89
wdi gdppc	0.82	0.69 – 0.99
Iexcluded_share>0TRUE	0.55	0.40 – 0.74
regime type	0.27	0.16 – 0.48
bs_t2directly_exposedduration_1	0.61	0.36 – 1.08
bs_t2directly_exposedduration_2	0.14	0.09 – 0.22
bs_t2directly_exposedduration_3	3.28	1.20 – 7.49
sds_t2directly_exposedduration_1	53299.43	28.90 – 15026988767.56
sds_t2directly_exposedduration_2	559.96	1.62 – 302397350.92
sds_t2directly_exposedduration_3	2154.25	2.13 – 349586876.94
Random Effects		
σ^2		1780141254272.75
τ_{00}		4914729502542.66
ICC		0.74
$N_{continent}$		4
Observations		1,434
Marginal R^2 / Conditional R^2		0.420 / 0.483

Figure S2 shows the random effects across continents for the *full+ri+t2* model (A) and the *full+ri+rs* model (B1-3). The effect of being directly exposed is zero in America for the *full+ri+rs* model, while the duration effect is much larger there than in the other continents. One reason for this could be that the spatial overlay-based measure of population exposure is less well able to capture variation in real impacts of flooding on people in the Americas, although it also could indicate problems with the displacement data (see also Figure 5 in the main article).

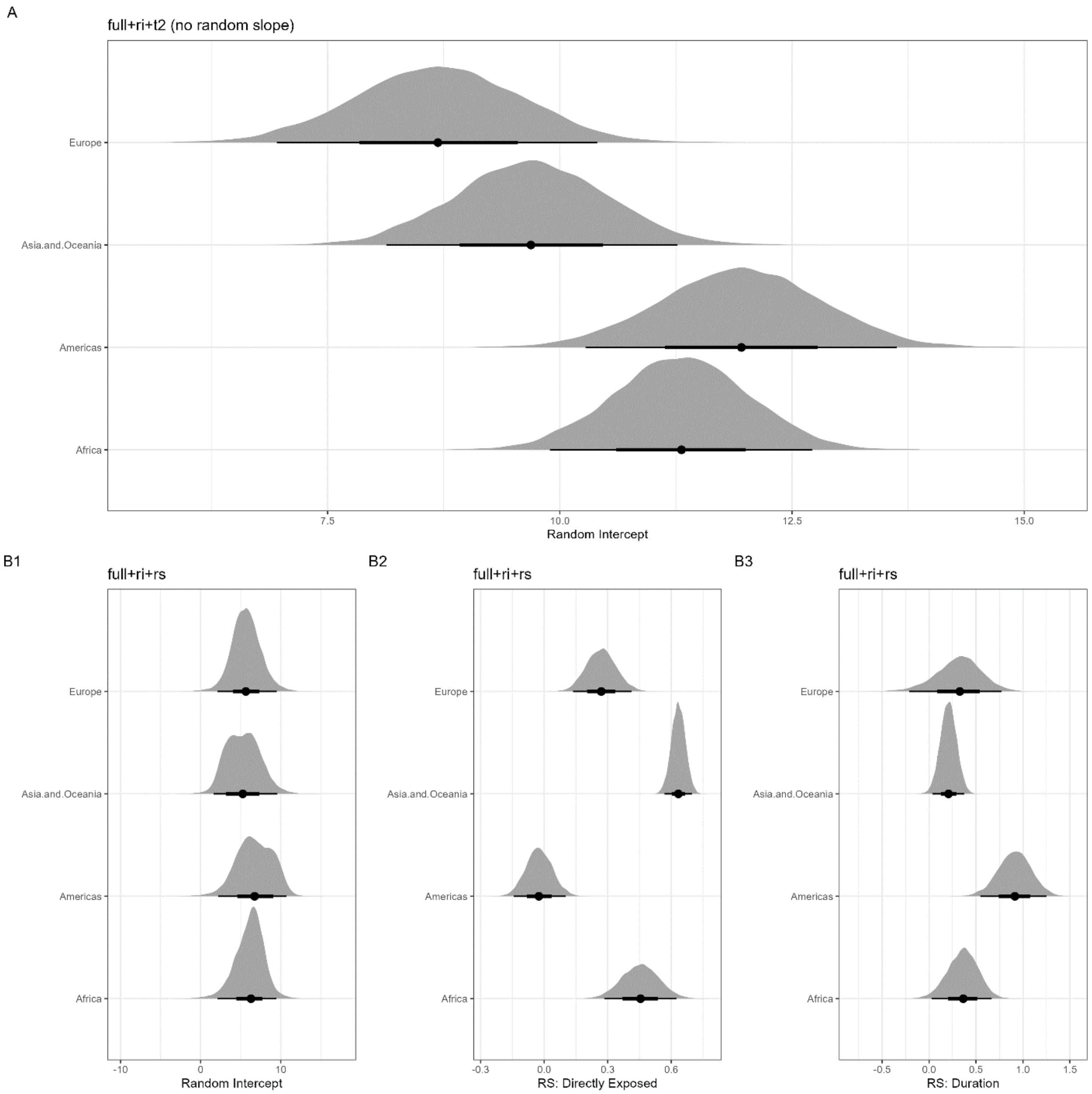

Figure S2: Random intercept and random slope across continents for two models

In the next two tables, we explore implications of only selecting the country-level (S9) or local (S10) indicator for each of the three contextual dimensions: socioeconomic and political development and conflict exposure. Both models include random intercept and tensor-interactions. Table S9 attains the highest R^2 of all estimated models, and where democracy in particular is an influential covariate. In this model, GDP per capita is not significant, contrasting the full model. Similarly, in the model with only local-level covariates (Table S10), political exclusion is not significant, while it is so in the full model.

Table S9: clvl+ri+t2

	**displaced w**	
Predictors	*Incidence Rate Ratios*	*CI (95%)*
Intercept	3384.73	245.62 – 33894.70
nevents sum 10	1.32	1.20 – 1.46
casualties brd sum 10	1.10	1.06 – 1.13
wdi gdppc	1.08	0.93 – 1.26
regime type	0.33	0.19 – 0.57
bs_t2directly_exposedduration_1	0.71	0.41 – 1.29
bs_t2directly_exposedduration_2	0.11	0.06 – 0.19
bs_t2directly_exposedduration_3	3.74	1.34 – 9.05
sds_t2directly_exposedduration_1	27696.58	37.13 – 3112641782.84
sds_t2directly_exposedduration_2	823.27	1.96 – 225929550.80
sds_t2directly_exposedduration_3	7436.35	2.50 – 1955765803.40
Random Effects		
σ^2		8022046589532.17
τ_{00}		19286307180946.43
ICC		0.75
$N_{continent}$		4
Observations		1,434
Marginal R^2 / Conditional R^2		0.479 / 0.496

Table S10: llvl+ri+t2

	displaced w	
Predictors	*Incidence Rate Ratios*	*CI (95%)*
Intercept	1979.82	204.70 – 14542.51
nevents sum 10	1.53	1.39 – 1.69
brd 6 mb	1.17	1.10 – 1.25
nlightsmean	1.36	1.18 – 1.57
Iexcluded_share>0TRUE	0.83	0.62 – 1.11
bs_t2directly_exposedduration_1	0.60	0.36 – 1.04
bs_t2directly_exposedduration_2	0.14	0.08 – 0.22
bs_t2directly_exposedduration_3	2.74	0.95 – 6.97
sds_t2directly_exposedduration_1	591018.70	788.98 – 135934133905.65
sds_t2directly_exposedduration_2	909.01	1.57 – 5955561352.91
sds_t2directly_exposedduration_3	291.35	1.32 – 56057306.35
Random Effects		
σ^2		1754770698272.52
τ_{00}		1591744417787.19
ICC		0.81
N $_{continent}$		4
Observations		1,434
Marginal R^2 / Conditional R^2		0.275 / 0.439

2.2. MCMC convergence

We have explored the convergence of our models through trace plots, the Rhat estimate, effective sample size, and reports of divergent transitions. We do get some (50-70) divergent transitions in the models with random slope, which could indicate that these models have not converged properly. The base, random intercept only, and the tensor interaction models do not have any (or only a couple) divergent transitions. Plots are made in the replication code.

We ran our models on 8 chains over 3,000 iterations with the first 1,500 being warm-up. This means we have 12,000 total post-warm-up draws for each of the 19 models. The target average proposal acceptance probability (adapt_delta) was increased to 0.99 to prevent Markov chain Monte Carlo (MCMC) convergence issues (divergent transitions). We used the same hyper-parameters for all models. Effective sample size and tail effective sample size is always well above (>2,500) the suggested 400 necessary to usefully diagnose Rhat. Rhat is always 1.00.

2.3. Exploring outliers

Outliers can affect the reliability of the performance estimates (PSIS-LOO) we use to compare predictive performance (both *elpd* and the stacking weights). We have explored predictive outliers by plotting the Pareto shape parameter k. Values larger than 0.5 can be regarded as outliers, and values above 1 are particularly problematic. The results from the main specification (Figure S3) are reassuring. The model with tensor interaction (Figure S4) have more outliers, meaning that the estimate of *elpd* is less trustful. For this reason, we prefer the *ri+rs* specification.

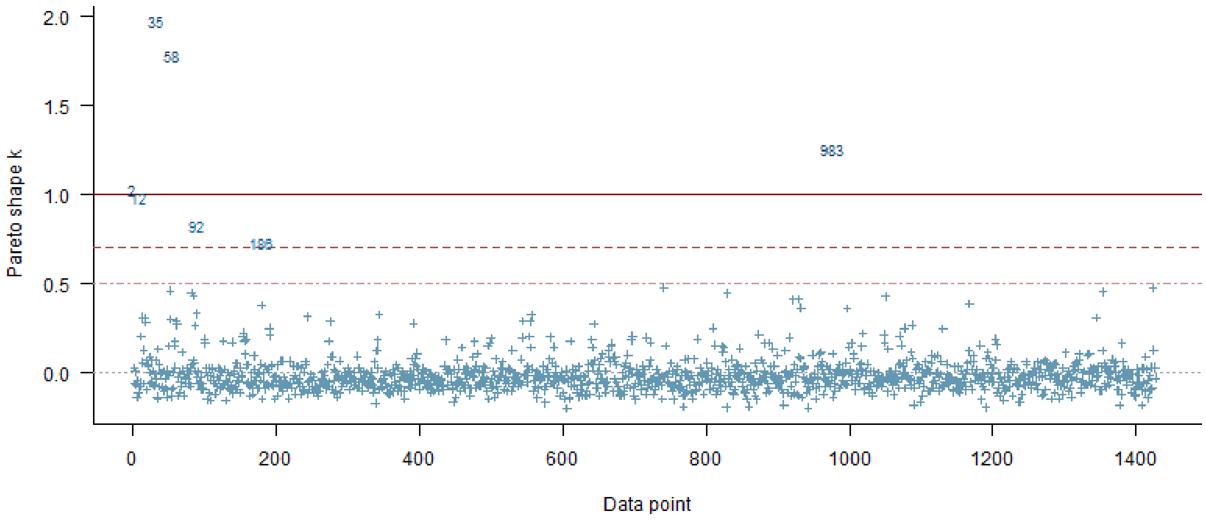

Figure S3: Predictive outliers and reliability of performance evaluation, full+ri+rs

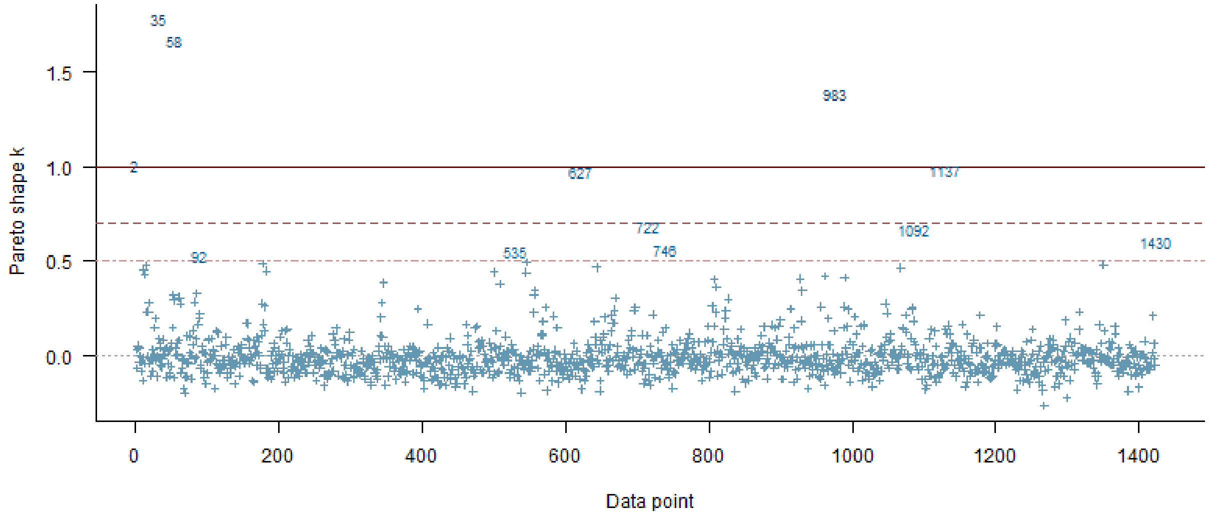

Figure S4: Predictive outliers and reliability of performance evaluation, full+ri+t2

2.4. Assessing predictive fit

As shown in Figure 4 in the main article, the *full+ri+rs* model is fairly well calibrated to the in-sample outcome. It tends to underpredict on lower levels of displacement and overpredict on the high end. The models with tensor spline interaction instead of random slopes, on the other hand, underpredict in Asia (and Oceania) while overpredicting everywhere else (Figure S5).

Out-of-sample, the *full+ri+rs* model is less well calibrated, and mostly miss the target through overprediction (Figure S6). The model that fits best out-of-sample according to the stacking weights is the *clvl-ri+t2* model (Figure S7). This model underpredicted in-sample in Asia, and since the displacement numbers have declined substantially in recent years, it fits even better out-of-sample than in-sample.

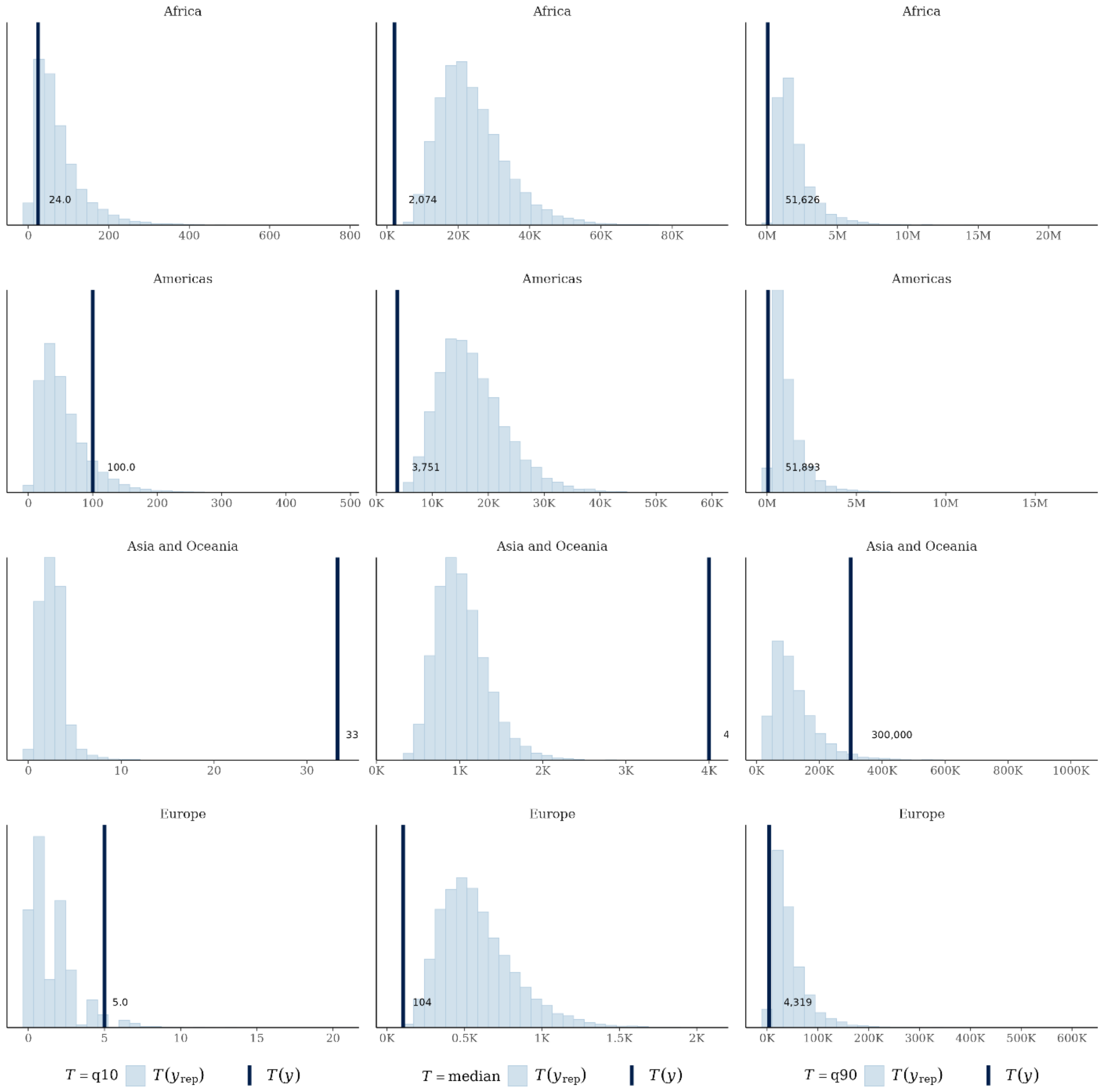

Figure S5: Predictive fit by continent, clvl+ri+t2

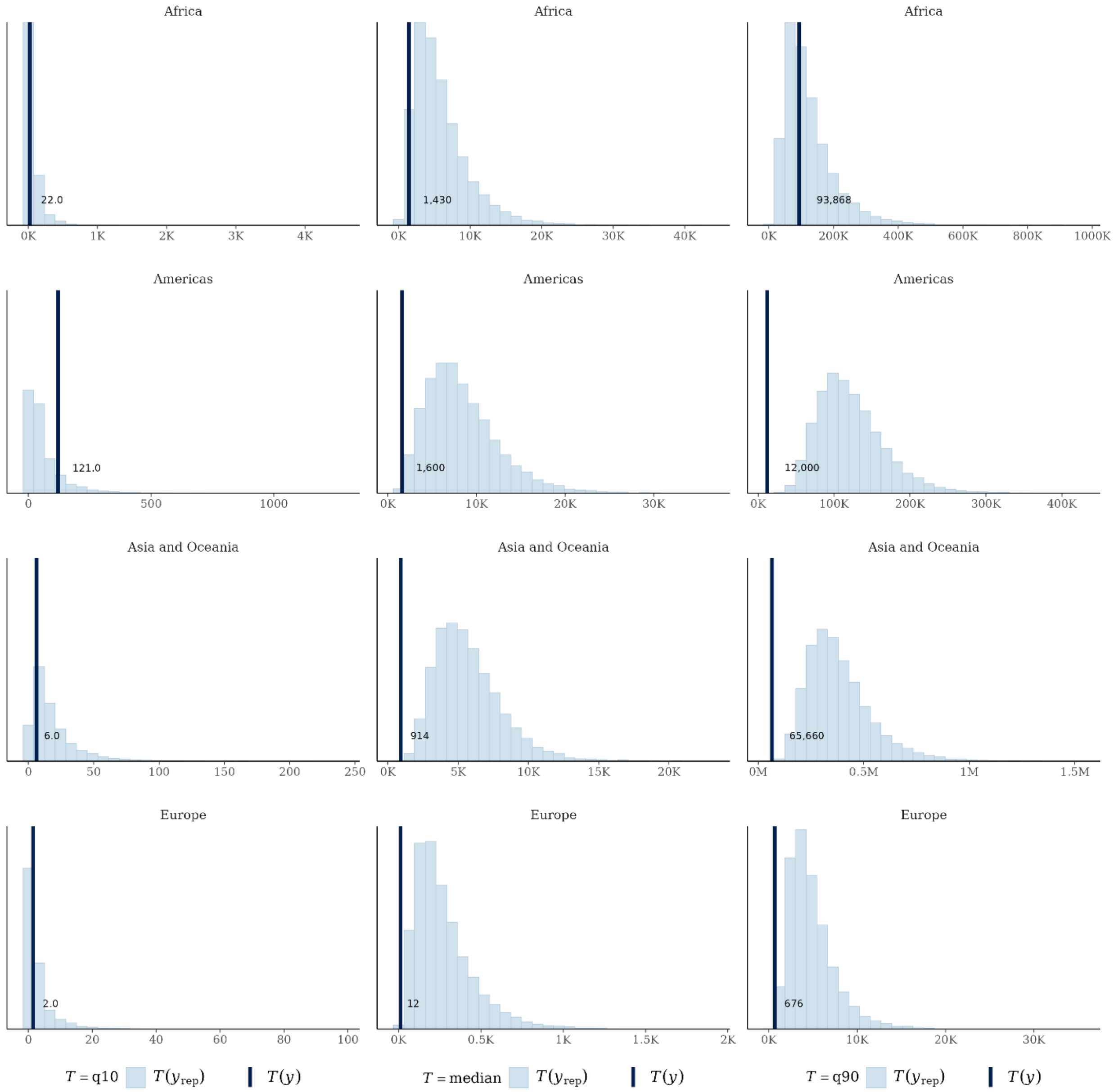

Figure S6: Predictive fit per continent, full+ri+rs, out-of-sample (2015-2018)

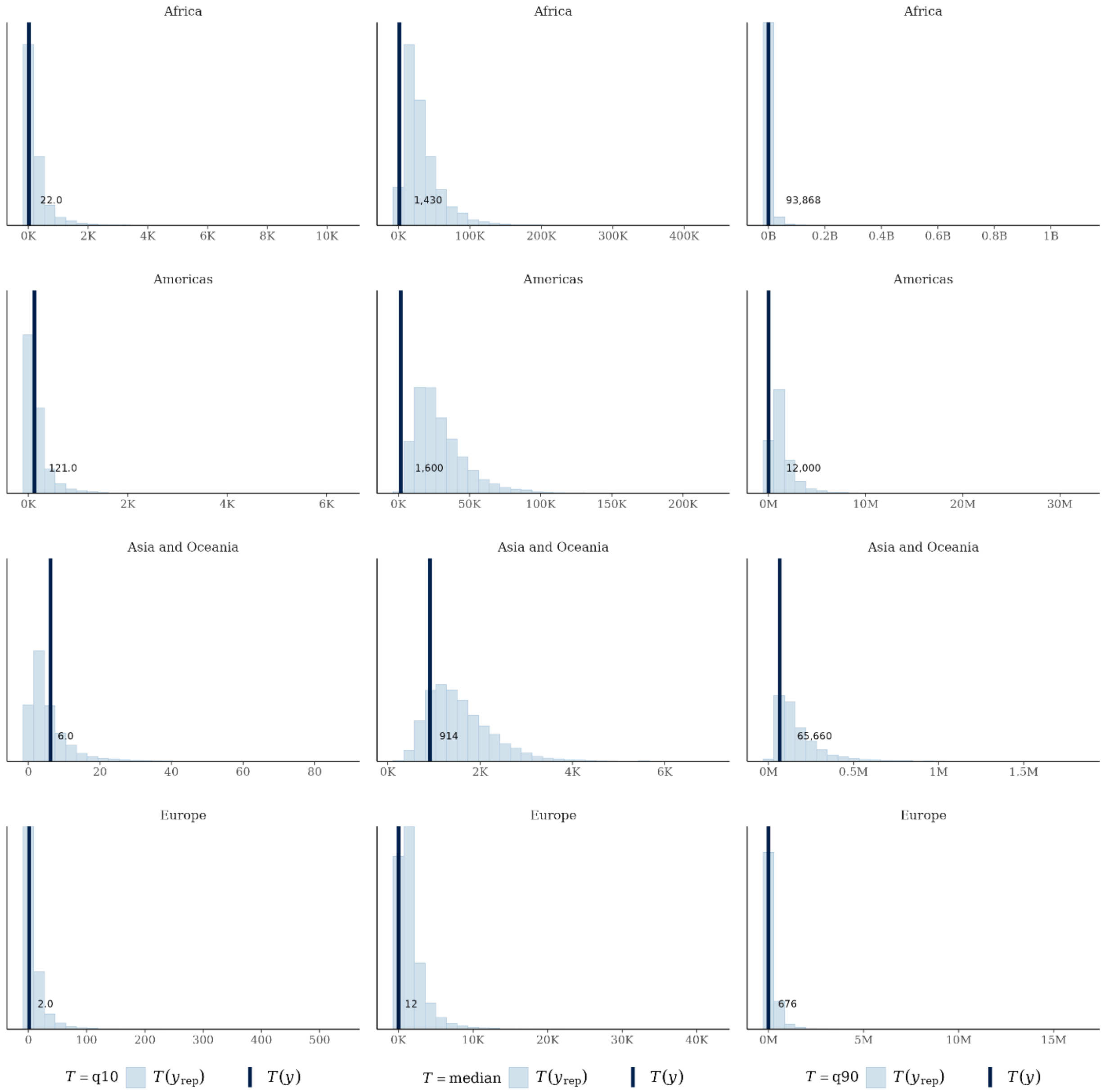

Figure S7: Predictive fit by continent, clvl+ri+t2, out-of-sample (2015-2018)

2.5. Model selection

While the results for the full models and corresponding stacking weights are shown in the main article (Table 1), here we focus on the individual and pairwise performance of the political, economic, and conflict contexts. Which sets of covariates are contributing the most to the predictive performance? The results are mixed as there is no clear correspondence between the best models in-sample and out-of-sample, except a tendency that simpler models work better out-of-sample (Table S13). While the economy-only model gets the strongest weight among the single element models in-sample, it is the conflict-only model that gets the strongest weight out-of-sample (Table S11). For the two-component models, the conflict + economy model gets the strongest weight, but conflict + politics is preferred out-of-sample (Table S12). Adding the covariates does not significantly improve the *elpd*.

Table S11: Predictive performance, comparing stacking weights in single theme models

**Predictive performance**						
**Model**	**In-sample (2000-2014)**			**Out-of-sample (2015-2018)**		
	**elpd LOO**	**Δelpd**	**Stacking W.**	**elpd LOO**	**Δelpd**	**Stacking W.**
**p+ri+rs**	-14424 (134)	0 (0)	0.2	-2222 (48)	0 (0)	0.31
**c+ri+rs**	-14425 (135)	-1 (14)	0.4	-2226 (49)	-4 (4)	0.35
**e+ri+rs**	-14426 (134)	-2 (14)	0.4	-2222 (47)	0 (3)	0.34

Table S12: Predictive performance, comparing stacking weights in dual theme models

**Predictive performance**						
**Model**	**In-sample (2000-2014)**			**Out-of-sample (2015-2018)**		
	**elpd LOO**	**Δelpd**	**Stacking W.**	**elpd LOO**	**Δelpd**	**Stacking W.**
**c-p+ri+rs**	-14407 (133)	0 (0)	0.35	-2227 (50)	-5 (8)	0.58
**e-p+ri+rs**	-14416 (134)	-10 (14)	0.23	-2223 (47)	0 (0)	0.42
**c-e+ri+rs**	-14419 (135)	-13 (18)	0.42	-2224 (48)	-1 (3)	0.00

Table S13: Predictive performance, comparing stacking weights in full and base models

**Predictive performance**						
**Model**	**In-sample (2000-2014)**			**Out-of-sample (2015-2018)**		
	**elpd LOO**	**Δelpd**	**Stacking W.**	**elpd LOO**	**Δelpd**	**Stacking W.**
**full+ri+rs**	-14395 (132)	0 (0)	0.77	-2226 (48)	-4 (4)	0.2
**base+ri+rs**	-14436 (136)	-41 (16)	0.23	-2222 (48)	0 (0)	0.8

Table S14 lists the in-sample and out-of-sample predictive performance of all 19 alternative specifications of models that estimate contextual determinants of flood-induced displacement.

Table S14: Predictive performance, including non-linear models (t2)

**Predictive performance**						
**Model**	**In-sample (2000-2014)**			**Out-of-sample (2015-2018)**		
	**elpd LOO**	**Δelpd**	**Stacking W.**	**elpd LOO**	**Δelpd**	**Stacking W.**
**full+ri+t2**	-14382 (131)	0 (0)	0.12	-2220 (47)	-1 (2)	0.00
**full+ri+rs**	-14395 (132)	-13 (19)	0.12	-2226 (48)	-7 (6)	0.00
**llvl+ri+t2**	-14403 (133)	-21 (12)	0.02	-2219 (46)	0 (0)	0.03
**clvl+ri+t2**	-14404 (132)	-22 (13)	0.13	-2219 (47)	0 (4)	0.39
**c-p+ri+rs**	-14407 (133)	-25 (21)	0.00	-2227 (50)	-9 (10)	0.01
**llvl+ri+rs**	-14410 (135)	-28 (25)	0.13	-2222 (48)	-4 (4)	0.17
**e-p+ri+rs**	-14416 (134)	-34 (23)	0.05	-2223 (47)	-4 (4)	0.00
**base+ri+t2**	-14419 (133)	-37 (18)	0.17	-2220 (47)	-1 (3)	0.08
**c-e+ri+rs**	-14419 (135)	-37 (26)	0.11	-2224 (48)	-5 (5)	0.00
**p+ri+rs**	-14424 (134)	-42 (24)	0.03	-2222 (48)	-3 (6)	0.17
**clvl+ri+rs**	-14425 (135)	-43 (26)	0.02	-2226 (49)	-7 (8)	0.00
**c+ri+rs**	-14425 (135)	-44 (26)	0.00	-2226 (49)	-7 (8)	0.05
**e+ri+rs**	-14426 (134)	-44 (26)	0.00	-2222 (47)	-3 (4)	0.00
**base+ri+rs**	-14436 (136)	-54 (27)	0.09	-2222 (48)	-3 (6)	0.00
**base+ri**	-14502 (143)	-120 (30)	0.00	-2219 (47)	-1 (3)	0.08
**base**	-14569 (149)	-187 (54)	0.00	-2226 (46)	-8 (4)	0.00
**clvl**	-14571 (154)	-189 (61)	0.00	-2227 (47)	-9 (5)	0.00
**full**	-14574 (155)	-192 (61)	0.00	-2229 (46)	-11 (5)	0.00
**llvl**	-14577 (152)	-195 (57)	0.01	-2227 (46)	-8 (4)	0.00

3. References

1. J. Vestby, S. Schutte, A. F. Tollefsen, H. Buhaug, Replication data for "Societal determinants of flood-induced displacement." *Harvard Dataverse* (2022) https:/doi.org/10.7910/DVN/JMAP2M.

2. G. R. Brakenridge, "Global Active Archive of Large Flood Events, 1985-Present" (DFO Flood Observatory, University of Colorado, 2021).

3. B. Tellman, *et al.*, Satellite imaging reveals increased proportion of population exposed to floods. *Nature* **596**, 80–86 (2021).

4. R. Sundberg, E. Melander, Introducing the UCDP Georeferenced Event Dataset. *Journal of Peace Research* **50**, 523–532 (2013).

5. T. Pettersson, *et al.*, Organized violence 1989–2020, with a special emphasis on Syria. *Journal of Peace Research* **58**, 809–825 (2021).

6. X. Li, Y. Zhou, M. Zhao, X. Zhao, A harmonized global nighttime light dataset 1992–2018. *Sci Data* **7**, 168 (2020).

7. World Bank, GDP per capita, PPP (constant 2017 international $) (2021).

8. M. Vogt, *et al.*, Integrating Data on Ethnicity, Geography, and Conflict: The Ethnic Power Relations Data Set Family. *Journal of Conflict Resolution* (2015) https:/doi.org/10.1177/0022002715591215.

9. M. Coppedge, *et al.*, "V-Dem Country-Year Dataset v11.1" (Varieties of Democracy Project, 2021).

10. C. R. Blyth, On Simpson's Paradox and the Sure-Thing Principle. *Journal of the American Statistical Association* **67**, 364–366 (1972).
